# The molecular dynamic simulation on impact and friction characters of nanofluids with many nanoparticles system

**DOI:** 10.1186/1556-276X-6-200

**Published:** 2011-03-08

**Authors:** Jizu Lv, Minli Bai, Wenzheng Cui, Xiaojie Li

**Affiliations:** 1 State Key Laboratory of Structural Analysis for Industrial Equipment, Department of Engineering Mechanics, Dalian University of Technology, Dalian 116024, China; 2 School of Energy and Power Engineering, Dalian University of Technology, Dalian 116024, China

## Abstract

Impact and friction model of nanofluid for molecular dynamics simulation was built which consists of two Cu plates and Cu-Ar nanofluid. The Cu-Ar nanofluid model consisted of eight spherical copper nanoparticles with each particle diameter of 4 nm and argon atoms as base liquid. The Lennard-Jones potential function was adopted to deal with the interactions between atoms. Thus motion states and interaction of nanoparticles at different time through impact and friction process could be obtained and friction mechanism of nanofluids could be analyzed. In the friction process, nanoparticles showed motions of rotation and translation, but effected by the interactions of nanoparticles, the rotation of nanoparticles was trapped during the compression process. In this process, agglomeration of nanoparticles was very apparent, with the pressure increasing, the phenomenon became more prominent. The reunited nanoparticles would provide supporting efforts for the whole channel, and in the meantime reduced the contact between two friction surfaces, therefore, strengthened lubrication and decreased friction. In the condition of overlarge positive pressure, the nanoparticles would be crashed and formed particles on atomic level and strayed in base liquid.

## Introduction

The concept of nanofluids is first introduced by Choi [1] from Argonne National Laboratory in 1995, which means the stable suspension engineered by suspending nanoparticles of metal, metallic oxide, or non-metallic with average sizes below 100 nm in base fluid. For it has superior heat transfer characteristics and would make remarkable improvement for heat transfer capability of heat exchange equipment, nanofluids has caused widely concerns in recent years. When nanoparticles are added into lubricating oils rather than traditional lubricant, the so-called "nano-lubricant" generates. There have been many investigations on the tribological properties of lubricants with different nanoparticles added [2-17]. The results with many experiments show that nanoparticles added to standard lubricating oils exhibit good friction-reduction and anti-wear properties.

The mechanisms of friction-reduction and anti-wear of nanoparticles in lubricant have been many researches [4-17]. Qiu et al. [4] found from their experiment that the tribological mechanism is that a deposit film in the contacting regions was formed, which prevented the direct contact of rubbing surfaces and reduced greatly the frictional force between the contacting surfaces. Chinas-Castillo and Spikes [5] investigated the mechanism of action of colloidal solid nanoparticles in lubricating oils. They found that in rolling contacts at slow speeds, colloids formed a boundary film of at least one or two times the particle size. Liu and Chen [6,7] have carried out studies on a wide range of different colloid solid nanoparticles using a four-ball tribotester. The results found that the deposition of tribochemical reaction products produced by nanoparticles during the friction process can result in an anti-wear boundary film, and decrease the shearing stress. Rapoport et al. [8-11] reported that the friction properties of the IF particles in oil were attributed to the following three effects: (a) the spherical shape of IF opens the possibility for an effective rolling friction mechanism; (b) the IF nanoparticles serve as spacer, which eliminate metal to metal contact between the asperities of the two mating metal surfaces; (c) third body material transfer. Wang et al. [12] investigated the tribological performance and anti-wear mechanism of Cu nanoparticles as liquid additives. The results found that nano-Cu additive can form a low shearing strength protection film in friction process, which has good self-repairing performance. Gu et al. [13] investigated anti-wearing and friction reducing mechanism of lubricating oils with nano-particles was discussed by adopting scanning electron microscope (SEM), energy dispersion spectrum (EDS), X-ray photoelectron spectroscopy (XPS), and atomic force microscope. The results found that nano-particles take the effect of the anti-wearing and friction reducing by the following five aspects such as "tiny polishing"; "tiny ball bearing," which can support loads; filling in and repairing worn surfaces; synergistic effect of big and small nano-particles and the new metal element and oxide film produced on the friction surface, which can protect the friction surface. Zhang et al. [14] investigated the tribological performance and anti-wear mechanism of Cu nanoparticles as lubricating oil additives. The results show that a deposit film containing metallic copper can form on the worn surface, which has a film thickness of about 120 nm. Peng et al. [15,16] found from their experiments that diamond and aluminum nanoparticle as additive in liquid paraffin at appropriate concentration can show better tribological properties for anti-wear and anti-friction than the pure paraffin oil. Scanning electron microscopy and energy dispersive spectrometer analyses can show that the thin films on the rubbing surfaces can be formed by these aluminum nanoparticles, which not only bear the load but also separate the both interfaces, thus the wear and friction can be reduced. Zhang et al. [17] investigated Cu nanoparticles as an oil additive and found at a load of 300 N a 4% additive amount of Cu nanoparticles exhibits the best self-repairing performance. Cu nanoparticles were deposited on the frictional surface to form deposited film during the friction process, which could get synergetic effect with the friction chemical reaction film coated on the surface.

Almost all research efforts on the mechanism of nanofluids lubricating property adopt experiment method. By means of friction testing machine, aiming at test workpiece surface, and utilizing SEM, EDS, and XPS technologies, the surface characteristics of different nanoparticles status could be obtained. The lubricated friction mechanism of nano lubricants could be estimated through analyzing testing results of test workpiece surface character. Therefore, most of present conclusions on lubricated friction mechanism of nano lubricants are experimental speculations, or rather, the essence of mechanism is still not clear.

In order to probe into the mechanism of nanofluids, molecular dynamics method has already been preliminary used. At present, the method is mainly used in research works on strengthen heat conduction [18-23] and flow characteristic of nanofluids [24-26], especially in the latter. Vergeles et al. [24,25] used molecular dynamics method and studied motor behavior of fluid in semi-infinite space and kinetic behavior when moved to walls. And the results confirmed the motions of nanoparticles could be figured by molecular dynamics method. Lv et al. [26] used molecular dynamics method and studied flowing behaviors of nanofluid constituted with liquid argon and copper nanoparticles between flat plates under shear flow conditions with different shearing velocities.

It follows that molecular dynamics method enables exact calculation on nanofluids. However, mechanism of shock and friction using nanofluids has not yet been investigated with molecular dynamics method. Thus, this study presents a molecular dynamics simulation on the essence characteristics of nanoparticle motions in enhanced lubrication and friction process, in order to explain the nature and mechanism of nanofluids in enhanced lubrication and friction.

In this study, impact and friction model of nanofluid for molecular dynamics simulation was built which consists of two Cu plates and Cu-Ar nanofluid. The Cu-Ar nanofluid model consisted of eight spherical copper nanoparticles with each particle diameter of 4 nm and argon atoms as base liquid. The motion states and interaction of nanoparticles at different time through impact and friction process could be obtained and friction mechanism of nanofluids could be analyzed.

### Simulation model and method

#### Numerical procedure

**Figure 1:**
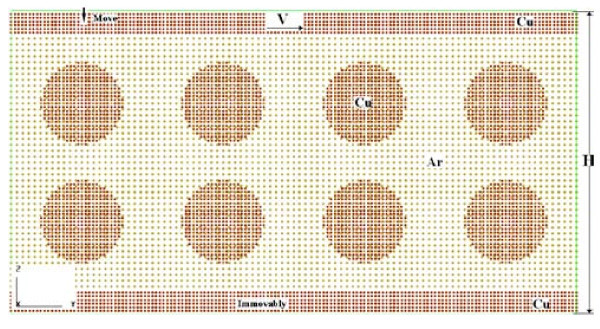
**The simulation model consists of two plates and nanofluids between them**. The nanofluids comprised eight Cu nanoparticles with the diameter of 4 nm and liquid Ar as base fluid. *H *is the height of the model, its initial value is 14.8 nm and it would change in impact process. The initial distance between two plates is 12.6 nm.

In this study, equilibrium molecular dynamics simulations are performed for nanofluids between two solid plates. As shown in Figure 1, the geometric model of the simulation cell has the size of 4.6 × 27.7 × 14.8 nm^3 ^and the distance separated between two plates is 12.6 nm. We adopted a base fluid model of argon, a nanofluid model of copper particles in argon and two solid plates model of copper. Although argon is not a real base fluid material used in experiments, it is the best choice for an initial nanofluids impact and friction molecular dynamics study.

To choose a suitable potential function is a crucial procedure to make sure the result is accurate and reliable in molecular dynamics simulation. However, currently there is no method rigidly accurate to describe the interactions between atoms or molecules. Therefore, empirical or semi-empirical correlations are adopted in most classic molecular dynamics simulation. Argon is chosen as base liquid on the basis of a well-defined potential function for it. For the widely accepted Lennard-Jones (L-J) potential matches experimental data for bulk fluid argon reasonably well, employs meaningful physical constants as parameters, and posses a simple, two-body form which requires much less computation time than more complex potentials involving other terms [21]. Various previous studies using molecular dynamics method on nanofluids properties have proved that the potential function could effectively indicate the intermolecular forces in nanofluids [18-23,26].

In this study, the interatomic interactions between solid copper, base liquid argon atoms and interactions between solid copper (Cu) and liquid argon (Ar) were all modeled by pairwise L-J potential [27] with appropriate L-J parameters,(1)

where *r_ij _*is the interatomic spacing between atoms *i *and *j*(*r_ij _*= *r_j _*- *r_i_*), ε and σ are parameters describing the bonding energy and bonding distance respectively. Though most accurate potential for modeling copper is embedded atom method (EAM) potential as it can also take care of metallic bonding, but in our present study L-J potential was used to reduce the computational time. To get the most quantitatively accurate results, more accurate EAM potential for that material should be used. However, since the aim of this study is to get the moving state and variation trend of nanoparticles in shock and friction process. Considering argon as the base fluid and modeling the interactions between copper atoms with L-J potential is a sensible choice. The bonding energy and bonding distance between copper and argon atoms are obtained according to Lorentz-Berthlot mixing law [28], which is given by(2)(3)

The LJ potential parameters of Ar-Ar, Cu-Cu, and Cu-Ar are shown in Table 1.

**Table 1:** LJ potential parameters for simulation

	σ (nm)	ε (J)
Argon (Ar)	0.3405	16.5402 E-22
Copper (Cu)	0.2338	65.5815 E-21
Cu-Ar nanofluid	0.2872	10.4153 E-21

#### Simulation model

The simulation model of nanofluids between two plates for molecular dynamics simulation was built by LAMMPS Molecular Dynamics Simulator, which consisted of liquid argon as base fluid and eight 4 nm copper nanoparticles.

The nanoparticle was sphere and prepared by carving from a copper cubic with initial FCC lattice arrangement. Then the nanoparticles were added into liquid argon cuboid, the overlapped liquid argon atoms were deleted. Each solid plate consists of six layers of copper molecules arranged as an FCC lattice. The whole simulation model has the total amount of 71,968 molecules, as shown in Figure 1.

The nanofluids is simulated by molecular dynamics simulation on a 4 core parallel computer in NTV ensemble at constant temperature of 86 K and the cutoff radius (*r*_cut_) is chosen to be 3*σ*_Ar_. Periodic boundary conditions are applied along the *x*- and *y*-directions and different asymmetrical boundary in *z*-axis direction are employed in different simulation cases.

The initial simulation system has a man-made atom distribution, so it needs to be relaxed adequately in order to allow the system to adapt itself to a more natural balance condition. In this study, it is relaxed for 600 ps with each time step length of 2 fs. The plates are fixed in the simulations.

**Figure 2:**
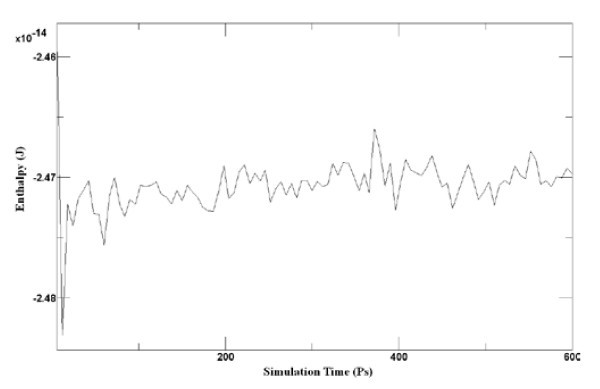
**Energy distribution in relaxation process**.

**Figure 3:**
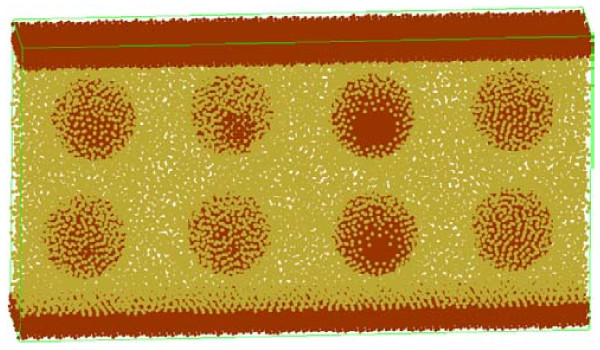
**The model ready for impact and friction simulation**.

The computer running time of relaxation takes about 24 h. And the energy distribution in relaxation process is shown in Figure 2. The enthalpy of system trends to converge which indicates the system reaches the equilibrium state. The relaxed model for impact and friction simulation is shown in Figure 3.

**Figure 4:**
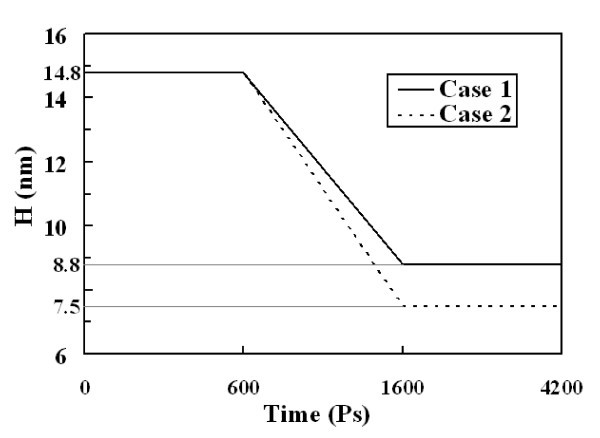
**Relationship between h and simulation time**.

After relaxation, symmetry boundaries are still applied along the *x*- and *y*-directions. From 600 to 4200 ps, the upper plate is given constant translational velocities of 100 m/s on *y*-axis and the lower one is still fixed to perform impact and friction simulation. As shown in Figure 1 both plates mutually compress. 600 to 1600 ps are for impact process and 1600 to 4200 ps are for friction simulation, respectively. Two cases have been designed to examine the effect of pressure. The only difference between them is that in case 1 H changes from 14.8 to 8.8 nm and in case 2 it changes to 7.5 nm. The length of time step is the same as relaxation, and the total computer running time during impact and friction simulation takes about 145 h. Figure 4 shows the relationship between h and simulation time.

From 0 to 600 ps is for relaxation, H keeps constant as 14.8 nm. From 600 to 1600 ps is for impact process, H changes from 14.8 to 8.8 nm and 7.5 nm in different cases, respectively. From 1600 to 4200 ps is for friction simulation and H keeps constant again in each case.

## Results and discussions

### Results discussion

**Figure 5:**
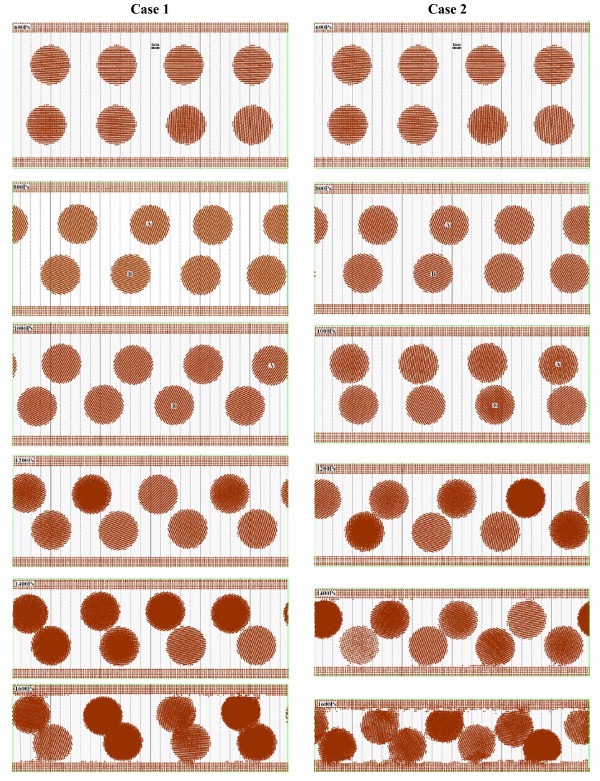
**Comparison of impact processes of two cases**. The screenshot times are at 600, 800, 1000, 1200, 1400, and 1600 ps.

**Figure 6:**
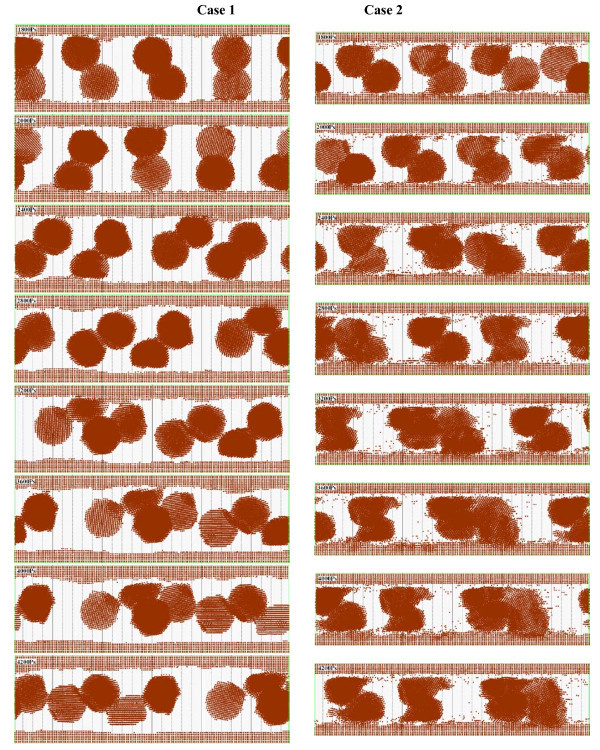
**Comparison of friction processes of two cases**. The screenshot times are at 1800, 2000, 2400, 2800, 3200, 3600, 4000, and 4200 ps.

The motion states of nanoparticles between plates in the processes of impact and friction under two compressed modes are shown in Figure 5. Through comparative analysis, it could be clearly observed that influenced by the strong shear force nanoparticles make translation motions between plates, in case 1 the velocity of nanoparticles in upper layer during 800 to 1000 ps is statistically estimated as 65.5 m/s, those nanoparticles in lower layer is 25.5 m/s; in case 2 the translation velocity of nanoparticles in upper layer is 55 m/s and that of nanoparticles in lower layer is 32 m/s; the velocity of nanoparticles in lower layer is much lower than that of nanoparticles in upper layer, and the main reason might be the absorption force of plate for the nanoparticles. And under different compressed modes, the shear translation velocities are different, the more pressure, the more obvious the effects for nanoparticles in the lower layer is, which is influenced by the internal flow with the external compression. In the meantime, it could be found that accompanying with the translation motion, the nanoparticles have drastic rotation. But with the compression process penetrating deeply, the rotation of nanoparticles is inhibited, and the reason might be that the interactions between nanoparticles are much stronger that the shear force by the upper plate, and thus, as influenced by the upper plate, the rotation effect is further reduced. Especially as the nanoparticles are interacting, the selection effect of nanoparticles is completely inhibited. In the compression process, the distribution of nanoparticles is affected to some extent, and the internal structure of nanoparticle would change. Under the effect of positive pressure from the upper plate, nanoparticles would be first absorbed to the plate, and then separate from it for the effect of the strong shear force; however, some metallic atoms from nanoparticles would remain being absorbed to the plate and made some filling effect to the plate. Figure 6 shows the motion state distribution of nanoparticles between plates in the friction process. In which it could be found that the nanoparticles formed apparent agglomeration, and with the increase of pressure, the agglomeration effect of nanoparticles is different, which shows that with higher pressure, the more obvious the agglomeration effect is. And the nanoparticles after agglomeration would serve as a supporting effect for the channel, and therefore, reduce the interactions between plates, strengthen the lubrication action, and decrease the friction. In addition, when the pressure is too high, nanoparticles would be crushed and some individual metallic atoms would stray in base liquid which has certain pollution effect to the lubrication system. And in the friction process, the aggregation of nanoparticles would move between the plates and interact with the plates, therefore make some metallic nanoparticles be adsorbed to the plate which supports a filling effect for the plates. Particularly for a rough surface, this absorption effect would make the surface smoother and decrease the frictional resistance further.

During impact process, nanoparticle made rotary motion and translational motion under effects of the shear force from plates before plates came into contact with nanoparticle. When coming into contact, plates would destroy the absorption layer first and then pressed nanoparticle. The transformation of nanoparticle depended on magnitude of impact force which is shown in Figure 5. In case 1, with lower impact force, nanoparticle merely had a small deformation, and with larger one in case 2, the nanoparticle was squashed and large deformation had been made. In the meantime of pressing nanoparticle, distribution of atoms in the plates near contact point was changed but would recover when the contact point had leaved nanoparticle. The impaction also cut some atoms in nanoparticle; these atoms would be absorbed to plates directly. Thus it had an effect of filling for rough surfaces. Figure 6 shows the comparison of friction processes of two cases after impaction. In case 1, nanoparticle still rotated mildly and enhanced friction process; in case 2, the nanoparticle was absorbed to the plates and became a part of it which could still improve surface-to-surface contact friction state. No matter how large the impaction force was, in later period, the destroyed absorption layer re-formed between surfaces of plates and nanoparticle and changed the interactions.

### Experimental verification

In the experimental work of Gu et al. [13] on friction mechanism of Cu nano lubricants, they have found traces of micro-buffing by nanoparticles on frictional surface and proved that spherical nanoparticles possess micro-ball effects through analysis of SEM patterns for test sample. This study simulated the shock and friction process of nanofluids, the micro-rotation motion of nanoparticles in the shock and friction process is visually observed by molecular dynamics simulations. Therefore, the previous experimental results are verified and the rationality of the present simulation is proved.

In addition, in pervious experimental works on friction mechanism, plenty of nanoparticles (some of them have already agglomerated) are observed to be absorbed to the friction surface which may have effects of filling and repair. In this study, aggregation of nanoparticles in shock and friction process is clearly observed. It could also be found that, in the shock process the nanoparticles would be absorbed to the friction surface. And through mutual effect, a nanomaterial protective film would form on the surface. The effect would be more obvious when surface is rough, and therefore, the AFM experiments have further verified the present simulation work is reasonable and effective.

## Conclusions

Model of nanofluids between two plates for molecular dynamics simulation was built which consisted of liquid argon as base fluid and eight 4 nm copper nanoparticles. L-J potential function was adopted to deal with the interactions between atoms. Through comparative analysis of simulation cases, the following conclusions were obtained.

1. Effected by the shear force, nanoparticles between two plates would make translation motion, and in the impact process, the nanoparticles also show violent rotation. But influenced by nanoparticles in the lower layer, in compression process the rotation of nanoparticles is restrained.

2. In the processes of impact and friction, nanoparticles would show obvious aggregation phenomenon, with the pressure increase, the effect of aggregation is more obvious. The aggregating nanoparticles would serve as a supporting effect for the plates and reduce the contact of two friction surfaces, strengthen lubrication, and decrease the friction effect. In addition, when the pressure is too high, nanoparticles would be crushed, and particles on atomic level would form and stray in base liquid.

3. During impaction, the argon absorption layer was first destroyed, and then plates and nanoparticle interacted. Nanoparticle would be pressed and some atoms from nanoparticle would be cut and absorbed to plates which had an effect of filling for rough plates, which would form a new nanoparticle protective crust and have an effect of filling for rough plates.

## Competing interests

The authors declare that they have no competing interests.

## Authors' contributions

JZL conceived of the study, carried out the molecular dynamics simulation and wrote the original paper. MLB and WZC performed the statistical analysis and revised the original manuscript. XJL participated in discussion of the results and in revising the manuscript. All authors read and approved the final manuscript.
